# Hydrophilic Carotenoids: Recent Progress

**DOI:** 10.3390/molecules17055003

**Published:** 2012-04-30

**Authors:** Magdolna Háda, Veronika Nagy, József Deli, Attila Agócs

**Affiliations:** Department of Biochemistry and Medical Chemistry, University of Pécs, Medical School, Szigeti út 12, H-7624 Pécs, Hungary

**Keywords:** carotenoids, hydrophilic, esterification, PEG conjugates, cycloaddition

## Abstract

Carotenoids are substantially hydrophobic antioxidants. Hydrophobicity is this context is rather a disadvantage, because their utilization in medicine as antioxidants or in food chemistry as colorants would require some water dispersibility for their effective uptake or use in many other ways. In the past 15 years several attempts were made to synthetize partially hydrophilic carotenoids. This review compiles the recently synthetized hydrophilic carotenoid derivatives.

## 1. Introduction

There are substantially two reasons for the synthesis of carotenoids with increased hydrophilic character: their possible use in medicine because of the altered or enchanced biological activity of the new compounds or their use in food (or feed) industry. Only a few hydrophilic carotenoids can be found in Nature, and among them the abundantly occuring crocin shows true water-solubility.

Several methods have been published (mostly patented) for enhancing the hydrophilicity of carotenoids. These formulations are typically obtained by physical methods, like polyethyleneglycol (PEG) dispersions. In this review only chemical derivatization including complexation is covered. The most important questions that arise in connection of the new derivatives—besides the extent of hydrophilicity—are whether they have at least the same antioxidant effect as the unmodified carotenoids and what are their toxicological and pharmacokinetic properties; the latter questions should be answered in the future for most of the following compounds. The antioxidant activity of some hydrophilic carotenoids increased in water when compared to the antioxidant activity of the hydrophobic parent compounds in organic solvents, as shown by Sliwka *et al.* [1].

## 2. Hydrophilic Salts of Carotenoid Esters

It seems quite obvious that transformation of a carotenoid into a hydrophilic derivative can be achieved by the synthesis of charged salt-like compounds or molecules that contain very polar groups (*i.e.*, the COOH groups of bixin or crocetin makes them at least slightly water-dispersible). In fact such compounds have been synthesized in the past few years, mainly by Lockwood *et al*. and Sliwka *et al*. 

**Figure 1:**
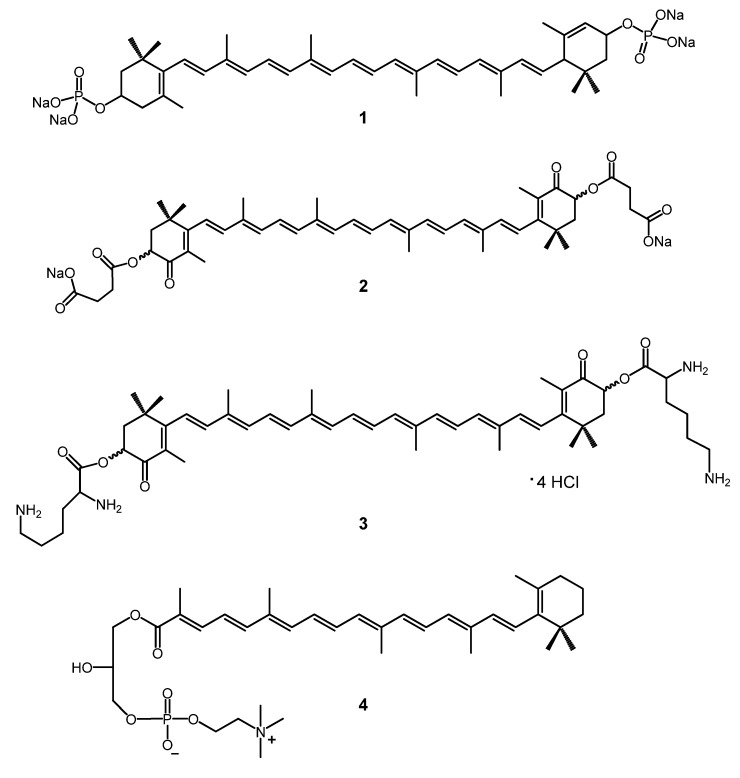
Hydrophilic carotenoid salts.

The astaxanthin disuccinate disodium salt **2** (Figure 1) [2,3], which has moderate water dispersibility, was one of the first hydrophilic carotenoid derivatives synthetized and it has already had a significant career as a powerful antioxidant. It is now in the clinical trials phase as a potential cardioprotective drug under the name Cardax [4,5,6]. Other derivatives like diphosphate **1** [7,8] and dilysinate **3** [9,10,11] are more hydrophilic for being more ionized (Figure 1). Compounds **1** (29.27 mg/mL) and **2** showed moderate water dispersibility (2.85 mg/mL), whereas **3** (181.6 mg/mL) is the first synthetic truly water-soluble carotenoid. Very recently Sliwka *et al*. synthetized some new cationic carotenoid lipids, where a quaternary ammonium moiety is responsible for the amphipatic character of the molecules [12,13].

Sliwka *et al.* also synthetized and studied thoroughly remarkable phospholipid derivatized carotenoids like **4** [14,15,16], and also investigated the physicochemical properties of natural hydrophilic carotenoids such as bixin and crocin [17,18,19]. Recently, they have synthetized aldoxime and ketoxime hydrochlorides from oxo carotenoids, which showed moderate water dispersibility [20]. The antioxidant and aggregation properties of all these compounds were extensively studied in the above mentioned articles [21].

## 3. Complexation with Cyclodextrins

Cyclodextrins (CD) are known biocompatible oligosaccharides of truncated cone shape, that have been applied in many fields of chromatography, environmental chemistry and also as food additives and complexing agents. Until the year 2000 there were no studies in connection with carotenoids that would reveal the real interaction between CDs and carotenoids. CDs were used as solubilizing agent, simply mixed with carotenoids in different proportions [22,23,24,25]. Later it was found that carotenoids without cyclic end groups at least on one end (e.g., lycopene or bixin) could form 1:1 inclusion complexes which are more stable than the native carotenoids [26,27,28,29,30,31]. We have found that cyclic end-groups cannot enter the relatively apolar cavity of CDs because of their size, but 3-4 CD molecules surround each end-group and are bound there through secondary interactions. A lot of CD derivatives were tested with various carotenoids and the most successful formulations were the randomly methylated β-CD (RAMEB) complexes of capsanthin, capsorubin and lutein [32]. The major drawback of these nanocapsulated carotenoids is that they contain a relatively high percent (95%) of CD which is required to maintain their complexation ability and the water solubility of the complexes. The aqueous solutions of these complexes are stable over months, so no aggregation can be observed, and the complexation is not pH dependent. The RAMEB-lutein complex has been recently found to facilitate the incorporation of lutein in neurons [33]. 

## 4. Glycosides

Carotenoid glycosides occur in Nature as constituents of cell membranes of certain heat-resistant microorganisms. These compounds, also called thermoxanthins, are slightly more hydrophilic than simple carotenoids, however, their amphiphilic structure is noteworthy. The length of the thermoxanthins is equal to the width of the phospholipid bilayer, and they are incorporated into the cell membrane modifying its properties. This particular behaviour of thermoxanthins is believed to be partially responsible for the heat resistance of the *Thermus* species [34].

Only a few methods have been published for the chemical synthesis of carotenoid glycosides: direct glycosylation of carotenoid alcohols using the classical Königs-Knorr procedure [35,36] and total synthesis starting from 3-hydroxy-β-ionone [37,38]. Glycosides of astaxanthin were also prepared by a biosynthetic process [39]. 

**Scheme 1:**
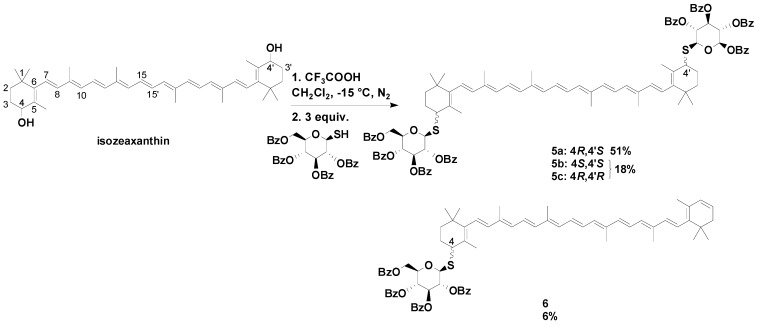
Synthesis of thermoxanthin mimetics.

Mimetics of natural thermoxanthins were prepared by the generation of dications from β-carotene or isozeaxanthin, which were treated with appropriate sugar derivatives as nucleophiles to make mono- and dithioglycosides [40]. The most successful reaction, to synthesize a thioglycoside in this case, can be seen in Scheme 1. After deprotection partially water-soluble thermoxanthin mimetics could be obtained. With this method CDs could be also coupled to carotenoids in the future.

**Figure 2:**
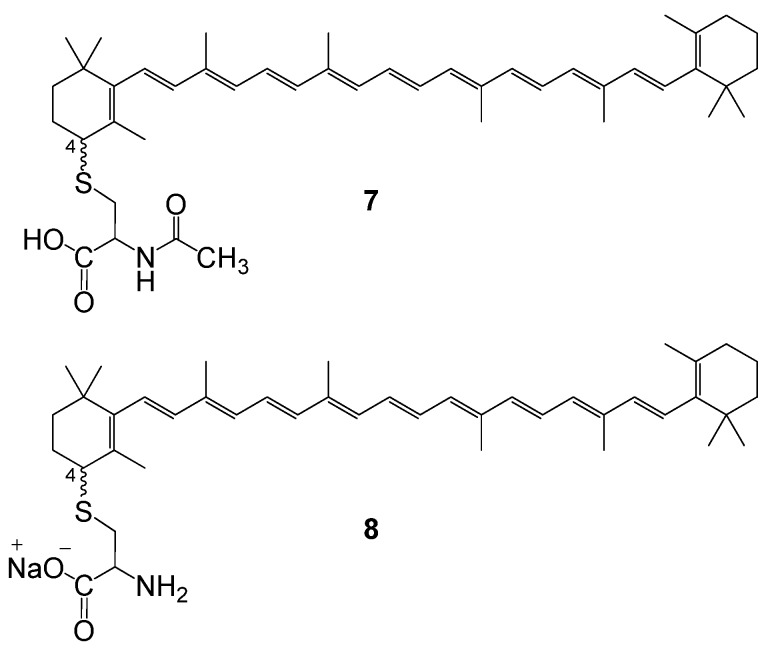
Cysteine conjugates of carotenoids.

In a similar way sulphur-containing amino acids can be coupled to carotenoids in good yields. With *N*-acetylcysteine as a nucleophile the products obtained are carotenoid-cysteine conjugates in which the amino acid moiety binds the carotenoid via sulphur (compound **7**, Figure 2). The water-dispersibility of the products can be increased by deprotection of the amino group to obtain compounds like **8** [41].

## 5. PEGylated Carotenoids

Polyethyleneglycol (PEG) conjugates of a wide range of biomolecules (especially peptides) are known [42,43], however, no covalently-bound PEG-carotenoid conjugates have been synthesized before. The hydrophilic PEG conjugates usually have better pharmacokinetic behaviour and, in general, are more efficient in drug targeting. There are examples of carotenoid-PEG dispersions in the literature which enhance the bioavailability of carotenoids [44]. PEG conjugates change the osmotic homeostasis much less than ionic compounds (such in those mentioned in Section 2). Furthermore, the water-solubility of PEG conjugates is independent of pH. If the PEG moiety is connected to the carotenoid through a relatively labile bond, which can be cleaved under physiological conditions, the PEG will serve solely as an indifferent, polar carrier for carotenoids. 

**Scheme 2:**
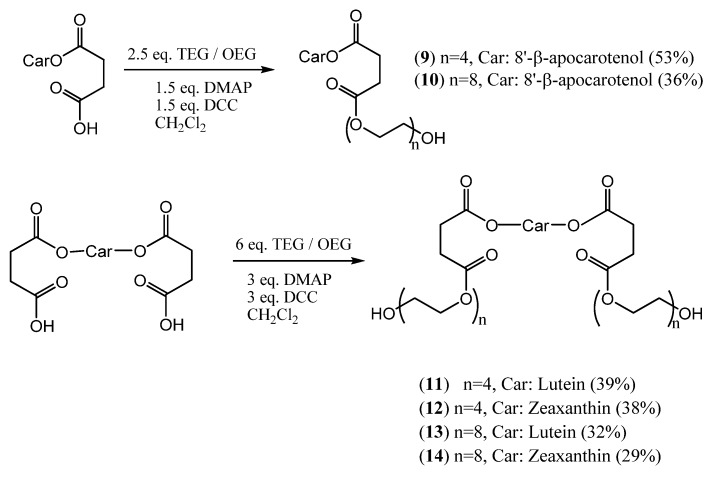
Synthesis of TEG- and OEG conjugates from mono- and disuccinates (Car = Carotenoid).

**Scheme 3:**
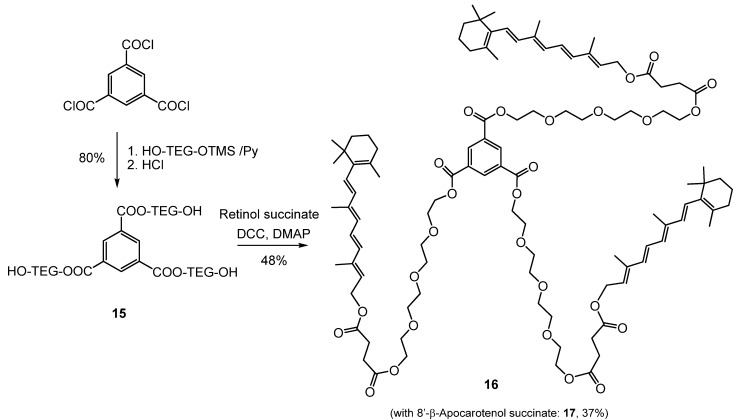
Trimers with OEG spacer.

Carotenoid-PEG esters and diesters were synthesized from several carotenoid succinates [45] with polyethyleneglycols of different chain length [tetraethyleneglycol (TEG), octaethyleneglycol (OEG), PEG-550 monomethyl ether (mPEG-550), Scheme 2] [46]. The same way carotenoid dimers and trimers that have a PEG spacer between the carotenoids were synthetized in good yields (Scheme 3) [47]. 

The water dispersibility of the products was compared and found, as expected, to be proportional with the PEG content of the conjugates. Although the conjugation via ester bond makes these compounds susceptible to hydrolysis (e.g., by pancreatic secretions), it might as well be an advantage that they are regenerated to the parent hydroxy-carotenoids under physiological conditions. Preliminary studies showed elevated antioxidant activity of some PEG-carotenoid ester conjugates (Scheme 2) in H_2_O_2_ induced oxidative stress. 

**Scheme 4:**
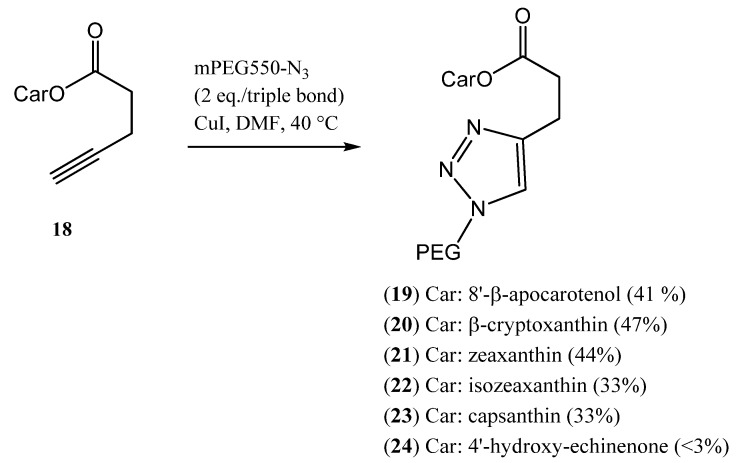
Synthesis of carotenoid-PEG conjugates via click-reaction.

Recently, we introduced azide-alkyne click chemistry to the field of carotenoid synthesis. After optimization of the reaction conditions, PEG azides could be coupled to carotenoid derivatives bearing an alkyne moiety [48]. This method seems to work well with carotenoids (Scheme 4), so it could be used in the future to synthesize not only carotenoid-PEG conjugates but conjugates with other, bioactive molecules.

## 6. Conclusions

Intensive research into hydrophilic carotenoid formulations of any kind commenced less than 15 years ago. In the past few years some attempts were made to make the carotenoids water dispersible via derivatization. As the example of Cardax shows, water-dispersible carotenoids can be more effective antioxidants and can display new properties in comparision with the parent carotenoids. Although some physiological and antioxidant studies were made on almost all of the compounds of this review, there is still a long way to go until the understanding of the action of hydrophilic carotenoids in contrast to native carotenoids.
